# Genome Sequence of a California Isolate of Fusarium oxysporum f. sp. *lycopersici* Race 3, a Fungus Causing Wilt Disease on Tomato

**DOI:** 10.1128/MRA.01713-18

**Published:** 2019-04-11

**Authors:** Peter M. Henry, Michelle Stueven, Sampson Li, Eugene M. Miyao, Thomas R. Gordon, R. Michael Davis, Hung K. Doan

**Affiliations:** a Department of Plant Pathology, University of California, Davis, California, USA; b University of California Cooperative Extension, Yolo County, Woodland, California, USA; University of Maryland School of Medicine

## Abstract

Fusarium wilt of tomato, caused by the soilborne fungus Fusarium oxysporum f. sp. lycopersici, is an increasingly important disease of tomato. This paper reports the high-quality draft genome assembly of F. oxysporum f. sp. *lycopersici* isolate D11 (race 3), which consists of 39 scaffolds with 57,281,978 bp (GC content, 47.5%), an *N*_50_ of 4,408,267 bp, a mean read coverage of 99.8×, and 17,682 predicted genes.

## ANNOUNCEMENT

Fusarium wilt of tomato, caused by the soilborne fungus Fusarium oxysporum Schlechtend.:Fr f. sp. lycopersici (Sacc.) W. C. Snyder & H. N. Hans., is a widespread and destructive disease in major tomato-growing regions worldwide (1). F. oxysporum f. sp*. lycopersici* comprises three races, based on pathogenicity to tomato cultivars carrying monogenic resistance genes (1, 2). We generated a high-quality draft genome assembly for F. oxysporum f. sp. *lycopersici* race 3 from California to serve as a reference for future studies of this pathogen. The isolate, D11, was obtained from a symptomatic plant in a commercial processing tomato field near Woodland, California, on 7 September 2010. D11 was isolated as a single spore from growth emerging from tissue cultured on Komada’s medium (3) and was found to be somatically compatible with tester strains representative of F. oxysporum f. sp. *lycopersici* VCG 0030 by the method described by Henry et al. (4). Race differentiation was confirmed by pathogenicity tests with the following tomato cultivars: Early Pak 7 (susceptible), VFN-8 (resistant to race 1), Walter (resistant to races 1 and 2), and CXD 282 (resistant to races 1, 2, and 3).

DNA was extracted from lyophilized conidia harvested from potato dextrose agar per Kaur et al. (5). Pacific Biosciences SMRTbell libraries were prepared at the University of California at Davis DNA Technologies Core, size selected for fragments larger than 15 kbp (BluePippin system), and sequenced with five single-molecule real-time (SMRT) cells on a PacBio RS II sequencing platform with P6C4 chemistry. A total of 396,897 reads (*N*_50_, 23,089 bp) passed quality filtering with polymerase read qualities above 0.80 and read lengths greater than 1,000 bp. These reads were assembled by HGAP3 (SMRT Analysis version 2.3.0) into 39 contigs with an *N*_50_ value of 4,408,267 bp and a mean read coverage of 99.8× (6). BUSCO (version 2.0) was used to assess completeness of the assembly as follows: 96.9% of expected complete, single-copy genes were found in the D11 assembly, which compared favorably to 94.8% identified in the isolate 4287 reference assembly (7). “Unitig_17” was identified as the mitochondrial genome sequence by comparison with a reference mitochondrial sequence (GenBank accession no. LT906324). Geneious (version R11.1) was used to self-align and circularize this sequence, starting with the “ATG” start codon from the *nad2* gene. CodingQuarry (8) was trained for gene prediction by mapping transcriptome sequencing (RNA-seq) reads (9) from the Spanish F. oxysporum f. sp. *lycopersici* 4287 to its corresponding reference assembly. With these training models, 17,682 genes were predicted in the D11 genome. Contigs were aligned to the F. oxysporum f. sp. *lycopersici* 4287 assembly to identify conserved chromosomes with progressiveMauve (10). Analysis of synteny showed multiple rearrangements in pathogenicity chromosome 14. A single G-to-C transversion at position 137 of the SIX3 (Avr2) gene was observed and is likely responsible for the lack of recognition by the *I-2* resistance gene of tomato (11).

### Data availability.

This whole-genome shotgun project has been deposited in DDBJ/ENA/GenBank under the accession no. RBXW00000000. The version described in this paper is the first version, RBXW01000000. Raw reads are available at NCBI GenBank under the accession no. SRR7892102.
